# RNA-seq analysis of extracellular vesicles from hyperphosphatemia-stimulated endothelial cells provides insight into the mechanism underlying vascular calcification

**DOI:** 10.1186/s12882-022-02823-6

**Published:** 2022-05-21

**Authors:** Zhong Peng, Yingjie Duan, Shuzhu Zhong, Juan Chen, Jianlong Li, Zhangxiu He

**Affiliations:** 1 The First Affiliated Hospital, Department of Gastroenterology, Hengyang Medical School, University of South China, Hengyang, Hunan 421001 China; 2 The First Affiliated Hospital, Department of Nephrology, Hengyang Medical School, University of South China, Hengyang, Hunan 421001 China; 3 School of Food and Biological Engineering, Hefei University of Technology, Hefei, 230002 China; 4 Department of Orthopedic Surgery, Nanfang Hospital, Southern Medical University, Guangzhou, 510515 China; 5 Department of Pediatrics, Weill Cornell Medicine, New York, NY 10021 USA

**Keywords:** Hyperphosphatemia, Extracellular vesicles (EVs), Vascular calcification, High-throughput sequencing (HTS)

## Abstract

**Background:**

Hyperphosphatemia (HP) is associated with vascular calcification (VC) in chronic kidney disease (CKD). However, relationship between HP-induced-endothelial extracellular vesicles (HP-EC-EVs) and VC is unclear, and miR expression in HP-EC-EVs has not been determined.

**Methods:**

We isolated HP-EC-EVs from endothelial cells with HP and observed that HP-EC-EVs were up-taken by vascular smooth muscle cells (VSMCs). HP-EC-EVs inducing calcium deposition was characterized by Alizarin Red S, colourimetric analysis and ALP activity. To investigate the mechanism of HP-EC-EVs-induced VSMC calcification, RNA-sequencing for HP-EC-EVs was performed.

**Results:**

We first demonstrated that HP-EC-EVs induced VSMC calcification in vitro. RNA-seq analysis of HP-EC-EVs illustrated that one known miR (hsa-miR-3182) was statistically up-regulated and twelve miRs were significantly down-regulated, which was verified by qRT-PCR. We predicted 58,209 and 74,469 target genes for those down- and up-regulated miRs respectively through miRDB, miRWalk and miRanda databases. GO terms showed that down- and up-regulated targets were mostly enriched in calcium-dependent cell–cell adhesion via plama membrane cell-adhesion molecules (GO:0,016,338, BP) and cell adhesion (GO:0,007,155, BP), plasma membrane (GO:0,005,886, CC), and metal ion binding (GO:0,046,914, MF) and ATP binding (GO:0,005,524, MF) respectively. Top-20 pathways by KEGG analysis included calcium signaling pathway, cAMP signaling pathway, and ABC transporters, which were closely related to VC.

**Conclusion:**

Our results indicated that those significantly altered miRs, which were packaged in HP-EC-EVs, may play an important role in VC by regulating related pathways. It may provide novel insight into the mechanism of CKD calcification.

**Supplementary Information:**

The online version contains supplementary material available at 10.1186/s12882-022-02823-6.

## Background

Vascular calcification (VC) is a pathological deposition of hydroxyapatite crystals in vascular wall, which is common in chronic kidney disease (CKD) [1]. As an independent predictor of cardiovascular events, VC is also significantly associated with mortality in CKD [2, 3]. There are multifactor (including metabolic disorders of serum phosphate and calcium, inflammatory cytokines, oxidative stress, and uremic toxins) accelerating VC in CKD patients, among which hyperphosphatemia (HP) is most strongly associated with VC [2]. It has been reported that endothelial extracellular vesicles (EC-EVs) can be released under HP circumstances [4]. However, there is rare information about the relationship between HP-EC-EVs and vascular smooth muscle cell (VSMC) calcification.

EC-EVs are formed from the outward budding of endothelial cells (EC) plasma membranes, and secreted to the extracellular chamber during stimuli such as HP [5, 6]. More and more researchers focus on the function of microRNAs (miRs) in EC-EVs. Several studies implicated that EC-EVs play an important role in inflammation, angiogenesis, and thrombosis by delivering miRs [7, 8]. There is a report showing that miR-126 and miR-26a in EC-EVs are significantly reduced in diabetic patients compared to non-diabetic patients [9]. Moreover, it has been demonstrated that miR-145-5p and miR-320a in EC-EVs could contribute to the progression of vasculitis [10]. Additionally, it has been reported that miR-29b, miR-133b, and miR-211 regulated VSMC calcification induced by HP [11]. Nevertheless, the different expression of miRs in HP-EC-EVs has not been determined.

In the current study, we firstly isolated HP-EC-EVs from endothelial cells treated with HP. We then observed the process of HP-EC-EVs being up-taken by VSMCs. The VSMC calcium deposition mediated by HP-EC-EVs was characterized by Alizarin Red S, quantified by colourimetric analysis and ALP activity. To better understand the mechanism of HP-EC-EVs-induced VSMC calcification in vitro, we detected the expression of miRs in HP-EC-EVs via high-throughput sequencing. Differentially expressed miRs (DEMs) from HP-EC-EVs, target genes of DEMs, and their related signaling pathway were performed by using bioinformatics analysis. The content of HP-EC-EVs may drive activation or down regulation of pathways related to vascular calcification.

## Methods

### Cell culture

The Human umbilical vein ECs (HUVECs) and VSMCs were obtained from the Type Culture Collection of the Chinese Academy of Sciences (Shanghai, China). The HUVECs were maintained in F12 K (Hyclone, Logan, Utah, USA) with 10% fetal bovine serum (Sigma, St. Louis, MA, USA) and 1% penicillin and streptomycin. To model hyperphosphatemia, NaH_2_PO_4_ was added to raise the [Pi] to 3.0 mM-a concentration that has been used extensively elsewhere [12, 13]. Pi concentration recorded was the concentration of the exogenous Pi. Medium harvested from experimental incubations was subjected to centrifugation as described below. The VSMCs were maintained in F12 (Hyclone, Logan, Utah, USA) with 10% fetal bovine serum and 1% penicillin and streptomycin. These cells were maintained under standard cell culture conditions of 37 °C, 5% CO_2_ and 95% humidity. The VSMCs were used for all experiments between passages 4 and 9.

### Isolation of EC-EVs

EC-EVs were isolated from the culture medium as previously described [4]. HP-EC-EVs were obtained by centrifugation of culture medium incubated with 3.0 mM Pi, while the control PBS-EVs were obtained with the same volume of PBS. Medium from cultures was centrifuged (step 1) at 1500 × g at 20 °C for 20 min to remove detached cells and large particles/apoptotic bodies. The top 90% of the supernatant from step 1 was centrifuged (step 2) at 14,000 × g at 20 °C for 30 min to pellet EVs. The top 90% of the supernatant from this step was aspirated, and the pellet was resuspended in the following 0.2-mm-filtered MP-buffer (145 mM NaCl, 2.7 mM KCl, and 10 mM Hepes, pH 7.4) and recentrifuged (step 3) as before to wash MPs before resuspending again in EV buffer and storing at -80 °C for additional analysis.

### Experimental procedures

Scanning electron microscopy, flow cytometry analysis of EC-EVs, EC-EVs labeling and uptake by VSMCs, in vitro calcification and quantification, Alizarin Red S, RNA extraction, miR sequencing library construction, high-throughput sequencing (HTS), bioinformatic analysis, and altered miR verification by qRT-PCR are described in detail in the Supplementary Materials and Methods.

### Statistical analysis

We examined the original data for normality and homogeneity of variance, and the data were expressed as the means ± standard deviation (SD). We analyzed the changes in EV count, calcium deposition levels, miRs PCR expression, and statistical calculations with SPSS 21.0 software (SPSS Inc., Chicago, IL, USA). *P* < 0.05 was considered to indicate a statistically significant difference. To analyze miR patterns in the PBS-EC-EVs and HP-EC-EVs groups, the miR counts were normalized using a modified global normalization method. Differential expression analyses were performed with the EdgeR package (3.18.1).

## Results

### VSMC calcification mediated by HP-EC-EVs

**Fig. 1:**
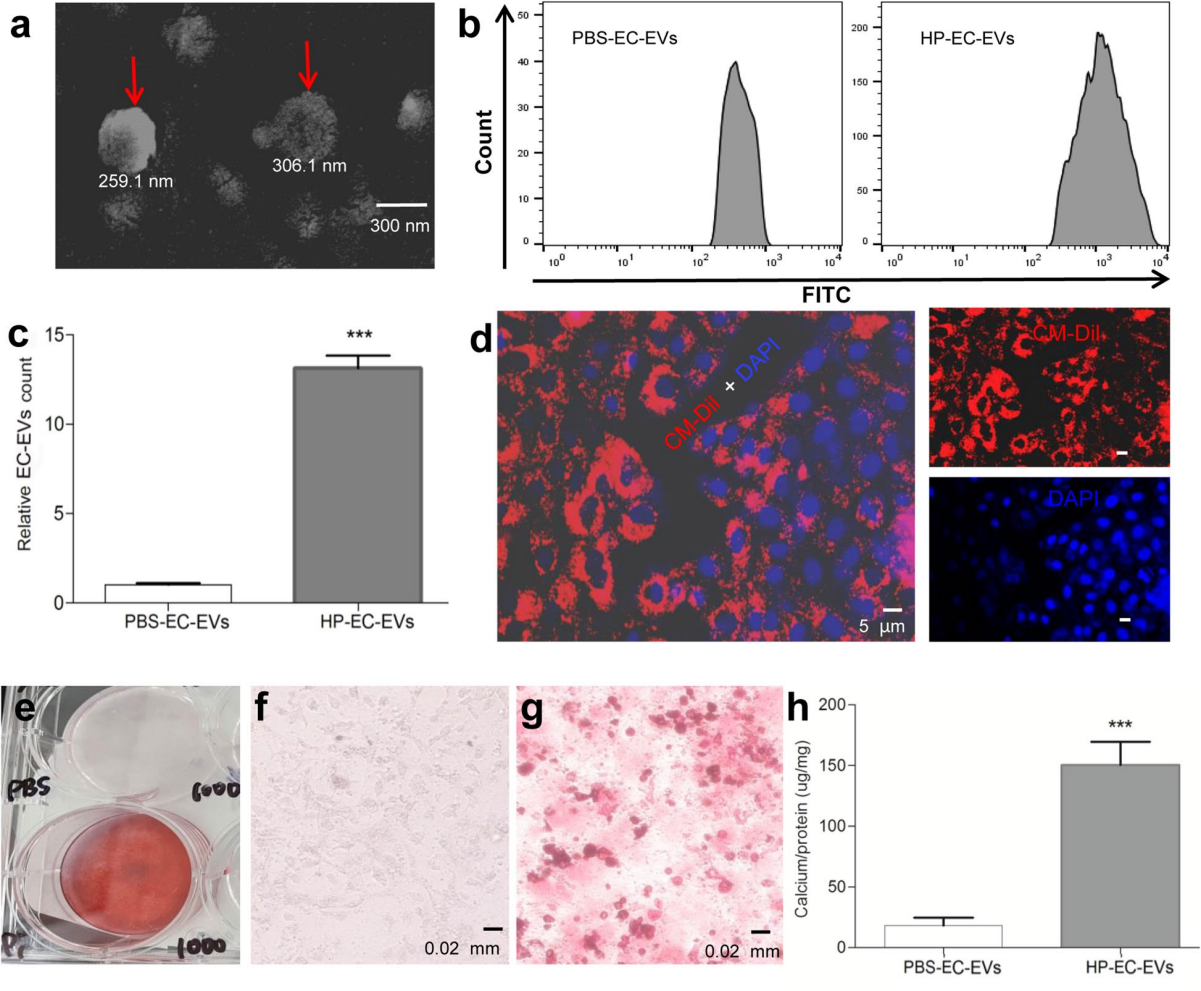
Endothelial cells exposed to HP promote the production of EC-EVs, and can induce VSMC calcification. **a** A representative electron microscopic image of EC-EVs isolated from the HUVECs cultured for 48 h in Pi (3 mmol/l) conditions. Scale bars: 300 nm. **b** A representative flow cytometry of HP-EC-EVs and PBS-EC-EVs. **c** The levels of EC-EVs in Pi (3 mmol/l) or PBS conditions for 48 h. Statistical significance was assessed using t-tests for two groups and is presented as follows: * or ^#^*p* < 0.05, ** or ^##^*p* < 0.01. ***p < 0.001. All values are means ± S.D. **d** VSMCs were incubated for 24 h with the 3 mM Pi-induced EC-EVs (HP-EC-EVs). HP-EC-EVs were stained with CellTracker CM-Dil (red). Nuclei were stained with DAPI (blue). Scale bars: 5 µm. A representative experiment from three different experiments is shown. **e** A representative experiment of **f** and **g** from three different experiments is shown macroscopically. Primary VSMCs were treated with **f** PBS-EC-EVs or **g** HP-EC-EVs (1000 µg/m) for 7 days, and the calcium deposition was analyzed by Alizarin red staining microscopically. Scale bars: 0.02 mm. **h** Total calcium in the lysates was measured and normalized by protein concentration. (*p < 0.05, **p < 0.01, ***p < 0.001 compared with control, each experiments were duplicated for three times)

We firstly isolated EC-EVs from the supernatants of HUVECs treated with 3 mM Pi (HP-EC-EVs). We showed the representative morphology of EC-EVs induced by HUVECs after 48 h treatment of 3 mM Pi (Fig. 1a) or PBS (Supplementary Fig. 1) detected by scanning electron microscopy. It showed that the mean diameter of the HP-EC-EVs was 282.6 nm. Our previous study showed that Pi stimulated EVs release from HUVECs for 48 h in a concentration-dependent manner [14]. Similar to the result, here, we detected a significant increase in HP-EC-EVs in comparison with PBS-EC-EVs by flow cytometry (Fig. 1b-c). Additionally, we observed that CM-Dil-labeled (red) HP-EC-EVs formed dots, rosettes, semicircle or circle around DAPI-labeled (blue) VSMCs (Fig. 1d). However, rare red could be found when PBS-EC-EVs were incubated with VSMCs (Supplementary Fig. 2).

To determine the ability of the HP-EC-EVs in regulating calcification, VSMCs were treated with HP-EC-EVs. After 7 days of the HP-EC-EVs (1000 µg/ml) exposure, VSMCs in HP-EC-EVs group exhibited more calcification deposition both macroscopically and microscopically by Alizarin Red S (Fig. 1e-g). Furthermore, calcium content was remarkably increased when VSMCs were exposed to the HP-EC-EVs (Fig. 1h).

### Different expression of miRs in HP-EC-EVs

**Fig. 2:**
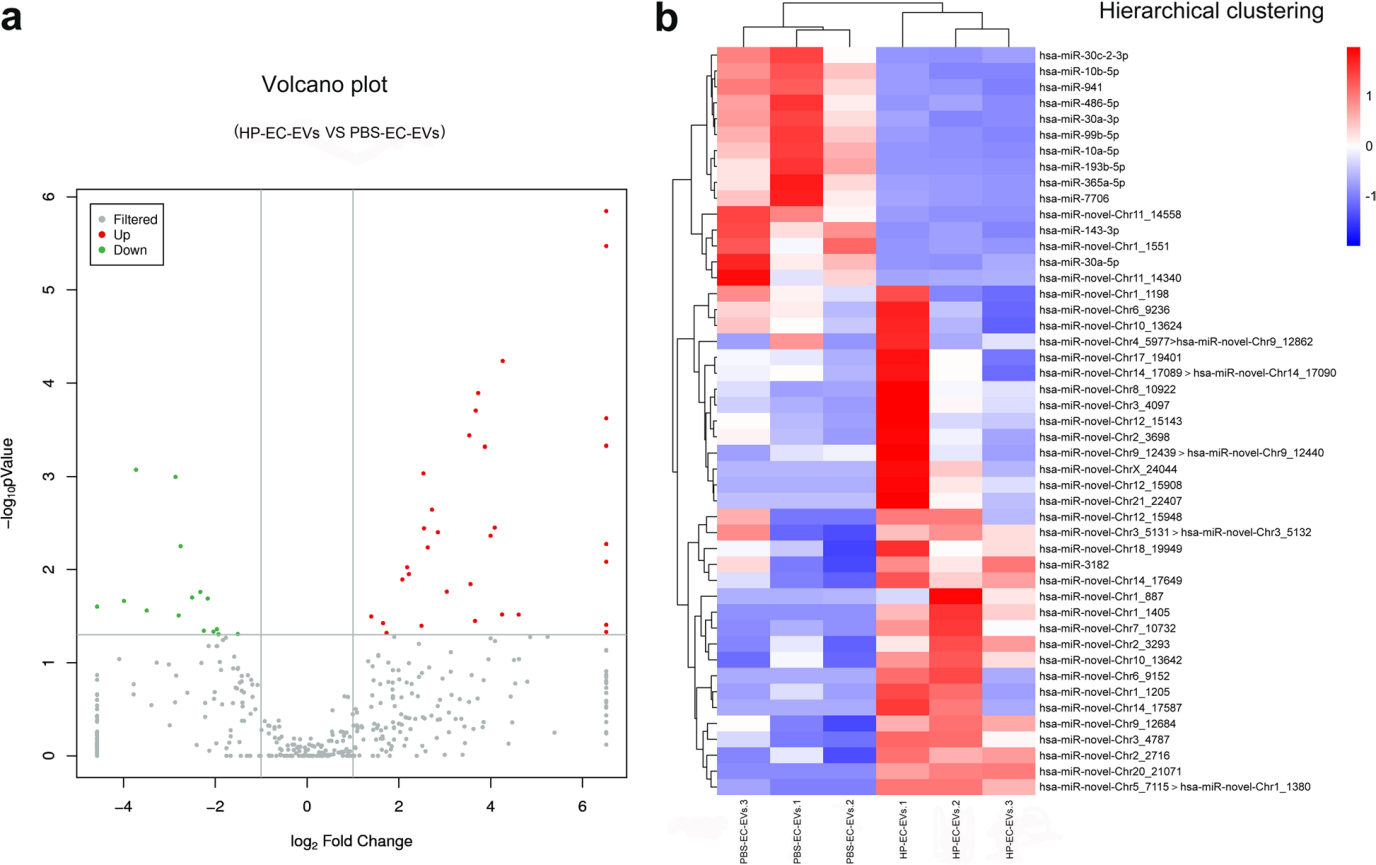
Clustering of expression patterns of 47 differentially expressed miRs. The expression patterns of 47 differentially expressed miRs (*P* < 0.05) in the 6 samples libraries are displayed in the volcano plot and clustering plots. Volcano plot **a** and clustering **b** of miRs in the HP-EC-EVs group and PBS-EC-EVs group. **a** Volcano plot was constructed using *P*-values and fold-change of miRs, with log (*P*-value) as the ordinate and log2 (Fold change) for the abscissa. Red and green dots represent differentially up- or down-expressed miRs between HP-EC-EVs and PBS-EC-EVs groups (*P* ≤ 0.05). **b** Hierarchical clustering was constructed according to the expression levels of miR, the six samples were classified into two groups. Blue represents low relative expression, and red represents high relative expression

From the six samples (three HP-EC-EVs and three PBS-EC-EVs), we examined 13 known miRs and 34 novel miRs differently expressed in HP-EC-EVs by next generation sequencing. There were twelve known down-regulated miRs and one known up-regulated miRs. There were three novel down-regulated miRs and thirty-one up-regulated novel miRs. Table S2 showed the differentially expressed miRs. Figure 2a and b showed the volcano and clustering plots of the differentially expressed miRs.

### Target gene prediction of differentially expressed miRs

**Fig. 3:**
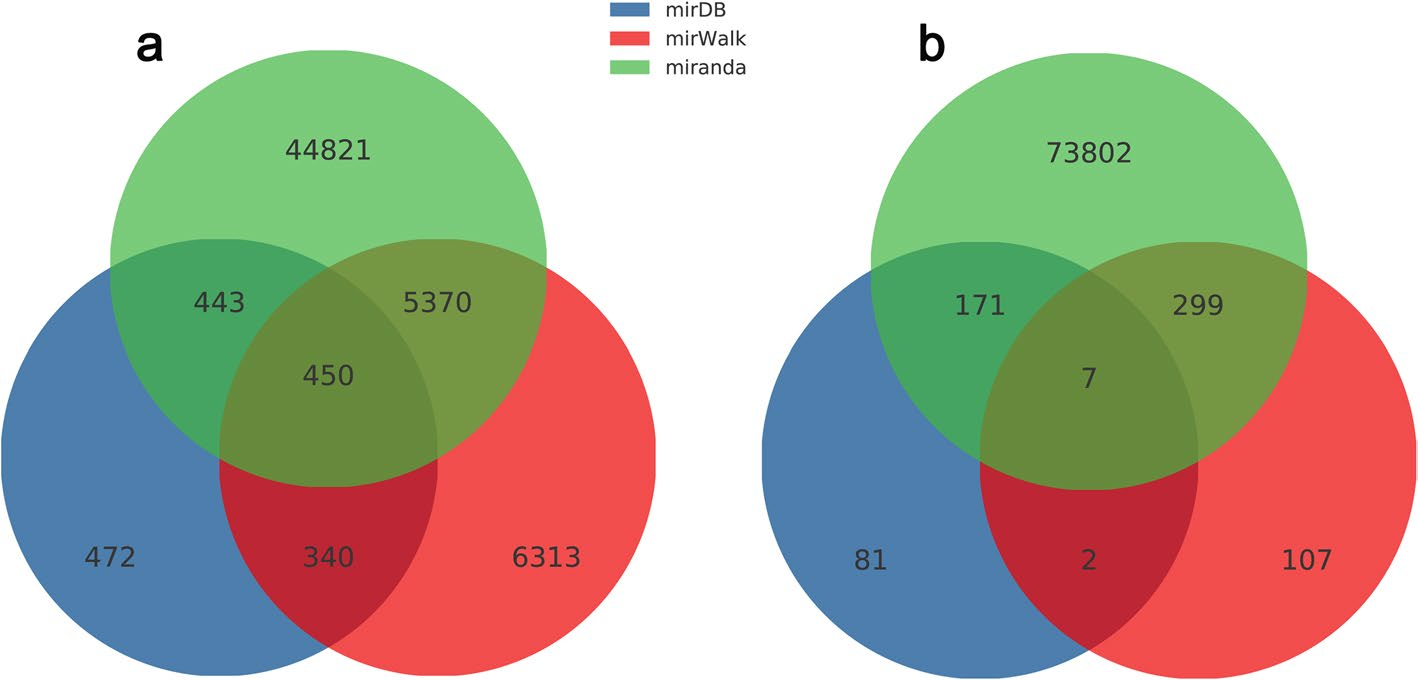
Venn diagram of differentially expressed miR target genes. Venn diagram showed the expression distribution of all the target genes of differentially expressed miRs. Numbers in parentheses represents numbers of co-expressed or differentially expressed miRs. **a** The Venn diagram of target genes in down-regulated miRs. **b** The Venn diagram of target genes in up-regulated miRs. Target gene prediction of the differentially expressed miRs was performed using miRDB, miRWalk and miRanda

We further analyzed the target gene prediction of the differentially expressed miRs via miRDB, miRWalk and miRanda databases. As the venn diagram showed in Fig. 3, there were 74,469 targets predicted in up-regulated miRs and 58,209 in down-regulated miRs totally. Our analysis showed 261 (miRDB database), 415 (miRWalk database), 74,279 (miRanda database), and 7 (common to the three databases) predicted target genes in up-regulated miRs, while 1705 (miRDB database), 12,473 (miRWalk database), 51,084 (miRanda database), and 450 (common to the three databases) predicted target genes in down-regulated miRs (Fig. 3a-b).

### Cytoscape network analysis of differentially expressed miRs

**Fig. 4:**
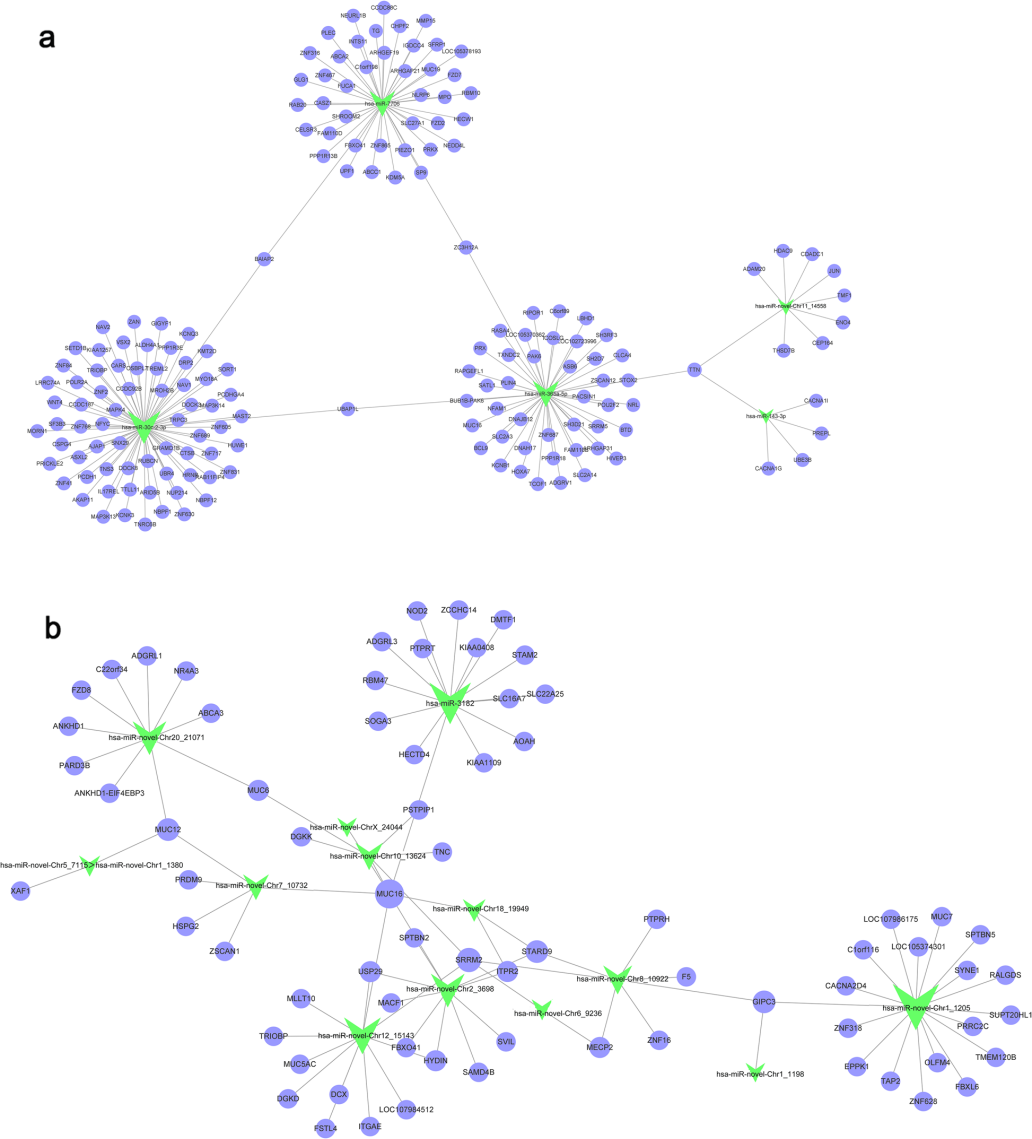
A proposed network of putative interactions between miRs and mRNAs. Green triangles represent down- or up-regulated miRs, and purple ellipses indicate the co-target genes. **a** miR-mRNA network among down-regulated miRs and their target mRNAs. **b** miR-mRNA network among up-regulated miRs and their target mRNAs

Figure 4 showed the cytoscape networks to illustrate the relationships between miRs differentially expressed in HP-EC-EVs and their target genes. Our analysis revealed that the miR-30c-2-3p, miR-7706, miR-365a-5p, miR-novel-Chr11_-_14,558, and miR-143-3p target genes were the most enriched and cross-linked networks in the down-regulated miRs (Fig. 4a). As for the up-regulated differently expressed miRs, the most enriched and cross-linked networks focused on the miR-novel-Chr1_-_1205, miR-novel-Chr12_-_15,143, miR-novel-Chr2_-_3698, miR-novel-Chr8_-_10,922, miR-novel-Chr18_-_19,949, miR-novel-Chr6_-_9236, miR-novel-Chr1_-_1198, miR-3182, miR-novel-ChrX_-_24044, miR-novel-Chr10_-_13,624, miR-novel-Chr20_-_21,071, miR-novel-Chr7_-_10,732,and miR-novel-Chr5_-_7115 > miR-novel-Chr1_-_1380 target genes (Fig. 4b).

### Gene ontology (GO) analysis

**Fig. 5:**
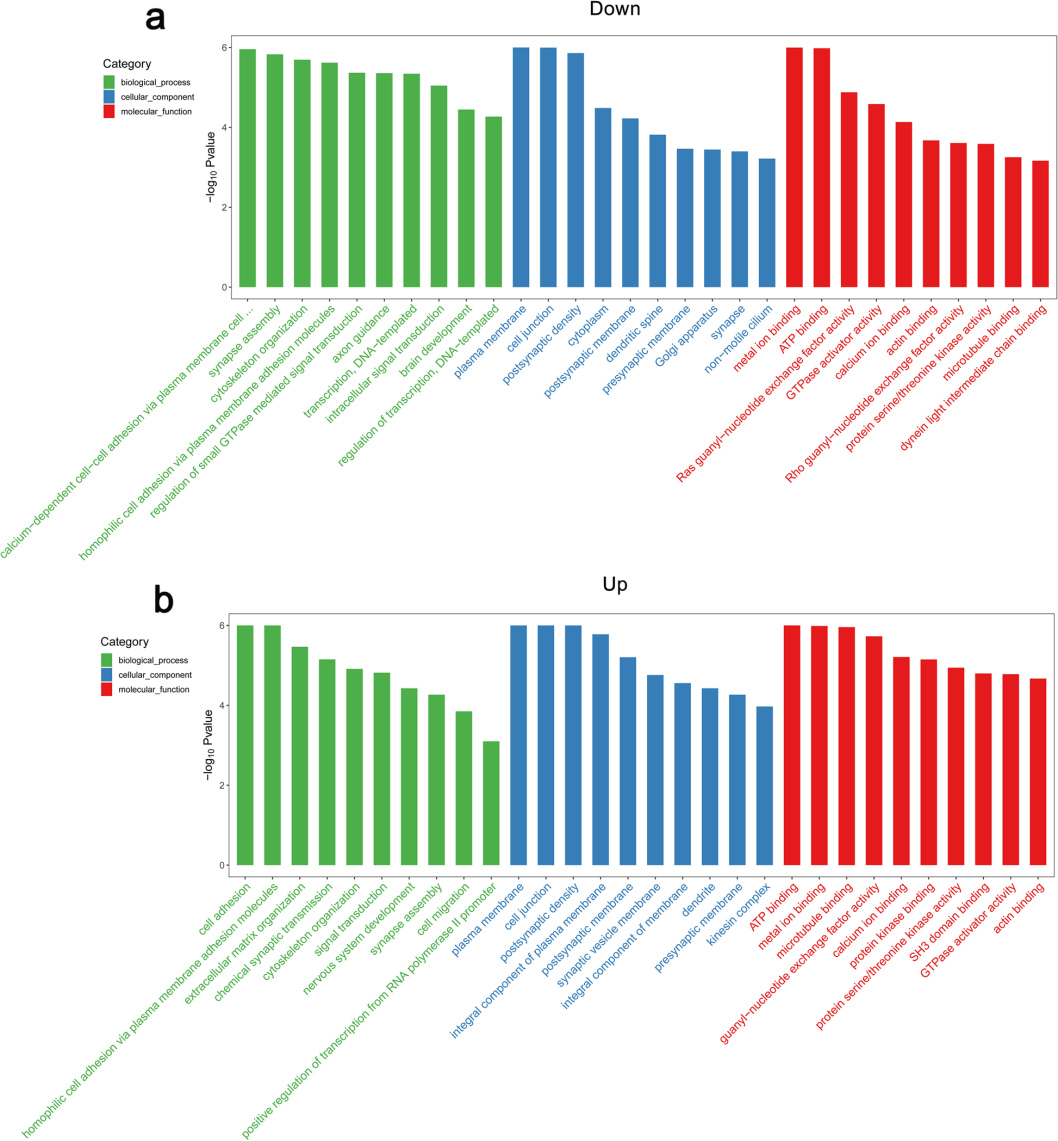
GO terms for DEMs. GO enrichment score [− log 10(*P*-value)] analysis of (**a**) down-regulated miRs and (**b**) up-regulated miRs. Top-10 GO terms are showed

Clustering GO analyses were performed according to target gene function of differentially expressed miRs (DEMs) from HP-EC-EVs and PBS-EC-EVs. It revealed entries enriched in biological processes (BP), cellular component (CC) and molecular functions (MF) of DEMs in HP-EC-EVs. There were 4895 GO terms enriched in the target genes of these significantly altered miRs. Figure 5 shows the top-10 GO terms for up-regulated and down-regulated miRs of the 3 ontologies (BP, CC and MF). Our bioinformatics analysis demonstrated that the down-regulated miRs were mainly enriched in calcium-dependent cell–cell adhesion via plama membrane cell-adhesion molecules (GO:0,016,338, BP), plasma membrane (GO:0,005,886, CC), and metal ion binding (GO:0,046,914, MF). As for the up-regulated miRs, they were enriched in cell adhesion (GO:0,007,155, BP), plasma membrane (GO:0,005,886, CC), and ATP binding (GO:0,005,524, MF).

### KEGG pathway analysis

**Fig. 6:**
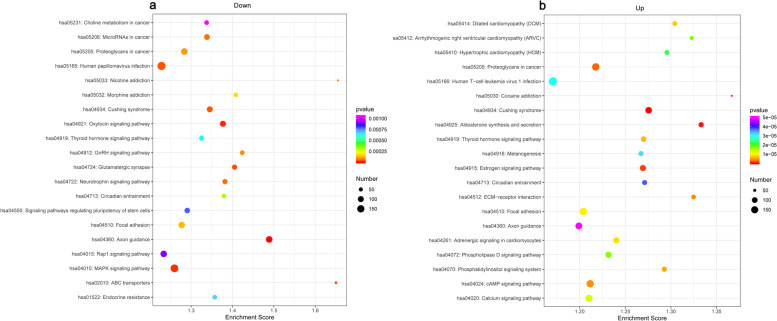
KEGG pathways of DEMs. KEGG pathways of differentially (**a**) down-expressed or (**b**) up-expressed miRs between HP-EC-EVs and PBS-EC-EVs groups

Our results showed that the down- and up-DEMs in the HP-EC-EVs groups participate in 320 and 321 pathways via KEGG pathway enrichment analysis. Supplementary Table S3 revealed the top-20 KEGG signaling pathways of total DEMs between HP-EC-EVs and PBS-EC-EVs groups. Autophagy signaling pathway (pathway ID: hsa04136) was the most significantly enriched pathways associated with total DEMs. However, ABC transporters (pathway ID: hsa02010) was a significantly enriched pathways associated with the down-regulated DEMs between HP-EC-EVs and PBS-EC-EVs group (Fig. 6).

### Novel miR prediction and verification of known miRs by qRT-PCR

There were 303 novel miRs in total in our current study. Among those novel miRs, we only found 3 down-regulated and 31 up-regulated differentially expressed novel miRs, based on the fold change (> 2 [up-regulated] or < 0.5 [down-regulated], *P* < 0.05). Supplementary Figs. 3 and 4 showed the predicted secondary structure maps of the differentially expressed novel miRs.

**Fig. 7:**
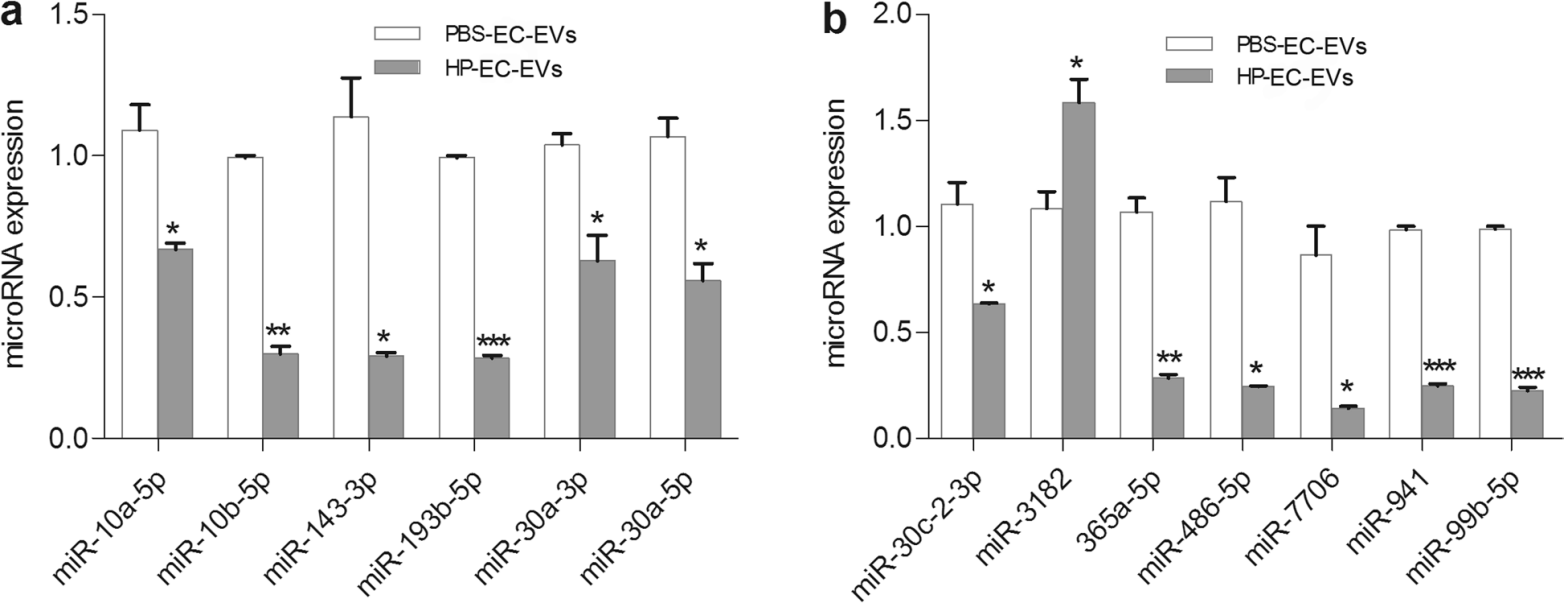
Verification of microRNAs (miRs) by qRT-PCR. **a** Significantly down-regulated miRs. **b** Significantly down- or up-regulated miRs. The results are expressed as the mean ± SD (three independent replicates per group). *p < 0.05, **p < 0.01, ***p < 0.001)

We further confirmed the known differently expressed miRs by qRT-PCR. The results showed that those 13 known miRs (including hsa-miR-10a-5p, hsa-miR-10b-5p, hsa-miR-143-3p, hsa-miR-193b-5p, hsa-miR-30a-3p, hsa-miR-30a-5p, hsa-miR-30c-2-3p, hsa-miR-365a-5p, hsa-miR-486-5p, hsa-miR-7706, hsa-miR-941, and hsa-miR-99b-5p) had relative lower levels in HP-EC-EVs group than PBS-EC-EVs group (*P* < 0.05). As for has-miR-3182, it had higher level in HP-EC-EVs group in comparison to that in PBS-EC-EVs (*P* < 0.05, Fig. 7).

## Discussion

There is increasing evidence that miRs play an important role in vascular calcification, and several researches have investigated the association among hyperphosphatemia, miRs in VSMC or its vesicles, and vascular calcification [11, 15]. However, there is rare information about the miR expression in HP-EC-EVs.

There are report that endothelial dysfunction can cause vascular calcification through BMP activation [16]. Hyperphosphatemia is a well-known factor that induces vascular calcification in CKD [17]. In the current study, our results indicated that HP-EC-EVs from injured endothelial cells might have the ability to induce VSMC calcification, and we observed the capture of HP-EC-EVs by VSMCs. Those captured HP-EC-EVs packaging miRs, might lead VSMCs calcification. Our results implicated that HP-EC-EVs and its cargos such as miRs potentially provide a link between the communication of activated endothelial cells and VSMCs to induce vascular calcification under hyperphosphatemia circumstances. Thus, we decided to explore the miR expression of HP-EC-EVs to better understand the CKD calcification.

RNA-seq and bioinformatics analysis were carried out to understand the different expression of miRs between HP-EC-EVs group and PBS-EC-EVs group. Those miRs (including hsa-miR-143-3p, hsa-miR-30c-2-3p, hsa-miR-30a-3p, hsa-miR-30a-5p, hsa-miR-486-5p, and hsa-miR-193b-5p) were associated with vascular calcification, and statistically down-regulated in HP-EC-EVs group. miR-143 is a potential biomarker of vascular calcification and cardiovascular disease associated with CKD, and it was down-regulated in VSMCs during the time course of Pi-induced vascular calcification [18–20]. Our current results showed that miR-143-3p was remarkably down-regulated in HP-EC-EVs. In consistent with our results, miR-143 expression in extracellular vesicles (EV) derived from urea and indoxyl sulphate-stimulated EC (EV^UR^) were also down-regulated, and mimicking of miR-143 in EV^UR^ blocked the pro-calcifying effects of EV^UR^ [21]. Among those miRs, the miR-30 family has been reported to play an important role in osteogenesis [22]. Our analysis revealed low level of miR-30c-2-3p in HP-EC-EVs. The published research that BMP-2 down-regulation miR-30c to increase Runx2 expression in human coronary artery SMCs and promoting mineralization may partly explain our result [23]. Additionally, the expression of miR-30a was significantly higher in VSMCs during vascular calcification [24–26]. However, miR-30a-3p and miR-30a-5p was significantly reduced in the HP-EC-EVs compared to controls in this study. This discrepancy may be due to miR different expression in VSMCs and HP-EC-EVs. Three novel miR (hsa-miR-novel-Chr11_14558, hsa-miR-novel-Chr11_14340, hsa-miR-novel-Chr1_1551) were also significantly down-regulated in HP-EC-EVs, but its structure and function remain elusive. Therefore, HP-EMP-induced VSMC calcification may be associated with the down-regulation of the abovementioned miRs.

In contrast, hsa-miR-3182 was markedly up-regulated in HP-EC-EVs. A recent research showed that hsa-miR-3182 and its host coding genes are genetically associated with cardiovascular disease [27], which supported our findings. Our analysis demonstrated that those target mRNAs for hsa-miR-3182 included NOD2, ZCCHC14, DMTF1, STAM2, SLC22A25, AOAH, KIAA1109, HECTD4, SOGA3, RBM47, ADGRL3, PTPRT, KIAA0408, SLC16A7, PSTPIP1. In consistent with our results, several lines of evidence demonstrated that NOD2 deficiency enhanced pulmonary VSMC proliferation, and exacerbated plaque necrosis in advanced atherosclerotic lesions [28, 29]. Furthermore, Sanneke et al. observed that SLC22A25 gene had strong signals associated with plaque morphology [30]. Additionally, RBM47 was identified to be associated with blood pressure or hypertension [31]. Taken together, target genes of hsa-miR-3182 may be associated with vascular calcification through regulation of blood pressure, VSMC proliferation, plaque morphology and necrosis. Surprisingly, only one up-regulated known miR was found while others were all belong to novel miRs. This remains us that we lack the understanding for those miRs, and further work about those differentially expressed miRs, especially those novel miRs, in HP-EC-EVs is still needed in the future.

In order to explore the regulatory mechanisms of differentially expressed miRs between HP-EC-EVs and PBS-EC-EVs, we annotated biological functions of miRs, predicted their targets and constructed regulation networks. This is the first study to identify a number of putative miR-mRNA interactions for DEMs in HP-EC-EVs (Fig. 4). GO enrichment analysis of DEMs in HP-EC-EVs is useful to describe BP, CC, and MF in relation to predicted target gene candidates.

In the current study, our top-20 KEGG pathway analysis revealed that calcium signaling pathway, cAMP signaling pathway, and ABC transporters were closely related to vascular calcification [32]. Early studies have suggested that roles for cellular calcium signaling involved in the regulation of calcification [33]. Integrative genomic study confirmed calcium signaling pathway genes RUNX2 and CACNA1C are associated with calcific disease [34]. Previous study has demonstrated that cAMP pathway promotes in vitro vascular calcification by enhancing osteoblast-like differentiation of calcifying vascular cells [35]. Later, Prosdocimo et al. reported that increased cAMP signaling and elevated extracellular inorganic phosphate (Pi) act synergistically to induce calcification of VSMC [36]. Thus, it is reasonable to understand that increased calcium signaling pathway or cAMP signaling pathway could be involved in the HP-EMP-induced VSMC calcification in vitro. ABC transporters represent a large family of ATP-driven transmembrane transporters involved in uni- or bidirectional transfer of substrates [37]. ABCC6, a unidirectional exporter protein, has been reported that its deficiency could alter ABC transporter gene expression and cause the ectopic mineralization disorder, characterized by calcification [37, 38]. Taken together, up-regulation of calcium signaling pathway or cAMP signaling pathway, and down-regulation of ABC transporters may be important in HP-EMP-induced VSMC calcification.

Surprisingly, except the vascular calcification-related pathways mentioned above, signaling pathways associated with cancer (such as Choline metabolism in cancer, MicroRNAs in cancer, and proteoglycans in cancer), endocrine system (such as Cushing syndrome, thyroid hormone signaling pathway, GnRH signaling pathway and endocrine resistance), nervous system (such as Rap1 signaling pathway, Axon guidance, signaling pathway regulating pluripotency of stem cells, and neurotrophin signaling pathway), and cardiomyopathy (such as dilated cardiomyopathy, arrhythmogenic right ventriculart cardiomyopathy, and hypertrophic cardiomyopathy) were also found in the HP-EC-EVs. Actually, EC-EVs have been reported to be a potentially useful biomarker of endothelial dysfunction in heart failure risk stratification [39]. However, there is rare information about the role of HP-EC-EVs in CKD or uremia patients. Our current study revealed that HP-EC-EVs may not only play important role in vascular calcification, but also in cardiomyopathy, nervous disease such as uremic encephalopathy, cancer, and endocrine disease in ESRD patients. This shed new light on the possible mechanism of several diseases in uremia patients, and further study about the potential role of HP-EC-EVs in those diseases especially for the cardio cerebrovascular disease leading high morbidity in CKD, should be explored in the future.

There are some limitations in this study. Our current study only focused on EVs induced by HP-ECs in vitro, which are not an arterial model. Although HP is important in VC, it is not the only factor and Pi binders is disappointed in the management of vascular calcification in CKD [40]. Thus, it is not clear whether these findings would apply to human disease. Considering the limitation of this study, further researches are absolutely needed to evaluate these findings, especially in vivo.

## Conclusion

This is the first study to perform miR-seq analysis in HP-EC-EVs. We identified 12 known down-regulated miRs (hsa-miR-10a-5p, hsa-miR-10b-5p, hsa-miR-143-3p, hsa-miR-193b-5p, hsa-miR-30c-2-3p, hsa-miR-30a-3p, hsa-miR-30a-5p, hsa-miR-365a-5p, hsa-miR-486-5p, hsa-miR-7706, hsa-miR-941 and hsa-miR-99b-5p) and 1 (hsa-miR-3182) known up-regulated miR. We also constructed miR-mRNA network, and performed GO term and related pathway analysis of the targets to predict the biological function of the altered miRs. We find that calcium signaling pathway, cAMP signaling pathway, and ABC transporters may have their special role in regulating vascular calcification. Our findings indicated that HP-EC-EVs might induce VSMC calcification and they do activate several pathways in the VSMCs. The differentially expressed miRs packaged in HP-EC-EVs might shed light on the mechanism of vascular calcification.

## Supplementary Information

**Additional file 1.**

**Additional file 2: Table S1.** The primer used in QPCR. **Table S2.** Down-regulated and up-regulated miRNAs. **Table S3.** KEGG pathways of total differentially expressed miRNAs between HP-EMPs and PBS-EMPs groups.

**Additional file 3.**

**Additional file 4.**

**Additional file 5.**

**Additional file 6.**

## Data Availability

The datasets used and/or analyzed during the current study are provided in the Supplementary Materials and Methods.

## Abbreviations

CKD: Chronic kidney disease
VSMCs: Vascular smooth muscle cells
Pi: Inorganic phosphate
HP: Hyperphosphatemia
EC-EVs: Endothelial extracellular vesicles
HP-EC-EVs: HP-induced-EC-EVs
ECs: Endothelial cell
HUVECs: Human umbilical vein ECs
VC: Vascular calcification
miRs: MicroRNAs
DEMs: Differentially expressed miRs

## References

[1] Paniagua-Sierra JR, Galván-Plata ME (2017). Chronic kidney disease. Rev Med Inst Mex Seguro Soc.

[2] Yamada S, Giachelli CM (2017). Vascular calcification in CKD-MBD: Roles for phosphate, FGF23, and Klotho. Bone.

[3] Mizobuchi M, Towler D, Slatopolsky E (2009). Vascular calcification: the killer of patients with chronic kidney disease. J Am Soc Nephrol.

[4] Abbasian N, Burton JO, Herbert KE (2015). Hyperphosphatemia, Phosphoprotein Phosphatases, and Microparticle Release in Vascular Endothelial Cells. J Am Soc Nephrol.

[5] Burger D, Schock S, Thompson CS (2013). extracellular vesicles: biomarkers and beyond. Clin Sci (Lond).

[6] Alique M, Ramírez-Carracedo R, Bodega G (2018). Senescent Microvesicles: A Novel Advance in Molecular Mechanisms of Atherosclerotic Calcification. Int J Mol Sci.

[7] Camaioni C, Gustapane M, Cialdella P (2013). extracellular vesicles and microRNAs: new players in the complex field of coagulation. Intern Emerg Med.

[8] McCarthy EM, Wilkinson FL, Parker B (2016). Endothelial extracellular vesicles: Pathogenic or passive players in endothelial dysfunction in autoimmune rheumatic diseases?. Vascul Pharmacol.

[9] Jansen F, Wang H, Przybilla D (2016). Vascular endothelial extracellular vesicles-incorporated microRNAs are altered in patients with diabetes mellitus. Cardiovasc Diabetol.

[10] Nakaoka H, Hirono K, Yamamoto S (2018). MicroRNA-145-5p and microRNA-320a encapsulated in endothelial extracellular vesicles contribute to the progression of vasculitis in acute Kawasaki Disease. Sci Rep.

[11] Panizo S, Naves-Díaz M, Carrillo-López N (2016). MicroRNAs 29b, 133b, and 211 Regulate Vascular Smooth Muscle Calcification Mediated by High Phosphorus. J Am Soc Nephrol.

[12] He J, Zhong X, Zhao L, Gan H (2019). JAK2/STAT3/BMP-2 axis and NF-κB pathway are involved in erythropoietin-induced calcification in rat vascular smooth muscle cells. Clin Exp Nephrol.

[13] Di Marco GS, Hausberg M, Hillebrand U (2008). Increased inorganic phosphate induces human endothelial cell apoptosis in vitro. Am J Physiol Renal Physiol.

[14] Xiang Y, Duan Y, Peng Z (2022). Microparticles from Hyperphosphatemia-Stimulated Endothelial Cells Promote Vascular Calcification Through Astrocyte-Elevated Gene-1. Calcif Tissue Int.

[15] Amabile N, Guérin AP, Leroyer A (2005). Circulating Endothelial extracellular vesicles Are Associated with Vascular Dysfunction in Patients with End-Stage Renal Failure. J Am Soc Nephrol.

[16] Davenport C, Harper E, Forde H (2016). RANKL promotes osteoblastic activity in vascular smooth muscle cells by upregulating endothelial BMP-2 release. Int J Biochem Cell Biol.

[17] Chen Y, Zhao X, Wu H (2020). Arterial Stiffness: A Focus on Vascular Calcification and Its Link to Bone Mineralization. Arterioscler Thromb Vasc Biol.

[18] Massy ZA, Metzinger-Le Meuth V, Metzinger L (2017). MicroRNAs Are Associated with Uremic Toxicity, Cardiovascular Calcification, and Disease. Contrib Nephrol.

[19] Louvet L, Metzinger L, Büchel J (2016). Magnesium Attenuates Phosphate-Induced Deregulation of a MicroRNA Signature and Prevents Modulation of Smad1 and Osterix during the Course of Vascular Calcification. Biomed Res Int.

[20] Rangrez AY, M'Baya-Moutoula E, Metzinger-Le Meuth V (2012). Inorganic phosphate accelerates the migration of vascular smooth muscle cells: evidence for the involvement of miR-223. PLoS ONE.

[21] Freise C, Querfeld U, Ludwig A (2021). Uraemic extracellular vesicles augment osteogenic transdifferentiation of vascular smooth muscle cells via enhanced AKT signalling and PiT-1 expression. J Cell Mol Med.

[22] Eguchi T, Watanabe K, Hara ES (2013). OstemiR: a novel panel of microRNA biomarkers in osteoblastic and osteocytic differentiation from mesencymal stem cells. PLoS ONE.

[23] Balderman JA, Lee HY, Mahoney CE (2012). Bone morphogenetic protein-2 decreases microRNA-30b and microRNA-30c to promote vascular smooth muscle cell calcification. J Am Heart Assoc.

[24] Ciavarella C, Motta I, Vasuri F (2021). Involvement of miR-30a-5p and miR-30d in Endothelial to Mesenchymal Transition and Early Osteogenic Commitment under Inflammatory Stress in HUVEC. Biomolecules.

[25] Vasuri F, Ciavarella C, Fittipaldi S (2020). Different histological types of active intraplaque calcification underlie alternative miR-mRNA axes in carotid atherosclerotic disease. Virchows Arch.

[26] Liu J, Xiao X, Shen Y (2017). MicroRNA-32 promotes calcification in vascular smooth muscle cells: Implications as a novel marker for coronary artery calcification. PLoS One.

[27] Zhu Y, Xie J, Sun H (2019). Three miRs cooperate with host genes involved in human cardiovascular disease. Hum Genomics.

[28] Kwon MY, Hwang N, Park YJ (2018). NOD2 deficiency exacerbates hypoxia-induced pulmonary hypertension and enhances pulmonary vascular smooth muscle cell proliferation. Oncotarget.

[29] Kwon MY, Hwang N, Back SH (2020). Nucleotide-binding oligomerization domain protein 2 deficiency enhances CHOP expression and plaque necrosis in advanced atherosclerotic lesions. FEBS J.

[30] Boer S, Baran Y, Garcia-Garcia HM (2018). The European Collaborative Project on Inflammation and Vascular Wall Remodeling in Atherosclerosis - Intravascular Ultrasound (ATHEROREMO-IVUS) study. Euro Intervention.

[31] Surendran P, Drenos F, Young R (2016). Trans-ancestry meta-analyses identify rare and common variants associated with blood pressure and hypertension. Nat Genet.

[32] Kanehisa M, Goto S (2000). KEGG: kyoto encyclopedia of genes and genomes. Nucleic Acids Res.

[33] Proudfoot D (2019). Calcium Signaling and Tissue Calcification. Cold Spring Harb Perspect Biol.

[34] Guauque-Olarte S, Messika-Zeitoun D, Droit A (2015). Calcium Signaling Pathway Genes RUNX2 and CACNA1C Are Associated With Calcific Aortic Valve Disease. Circ Cardiovasc Genet.

[35] Tintut Y, Parhami F, Boström K (1998). cAMP stimulates osteoblast-like differentiation of calcifying vascular cells. Potential signaling pathway for vascular calcification. J Biol Chem.

[36] Prosdocimo DA, Wyler SC, Romani AM (2010). Regulation of vascular smooth muscle cell calcification by extracellular pyrophosphate homeostasis: synergistic modulation by cyclic AMP and hyperphosphatemia. Am J Physiol Cell Physiol.

[37] Vanakker OM, Hosen MJ, Paepe AD (2013). The ABCC6 transporter: what lessons can be learnt from other ATP-binding cassette transporters?. Front Genet.

[38] Ibold B, Faust I, Tiemann J (2019). Abcc6 deficiency in mice leads to altered ABC transporter gene expression in metabolic active tissues. Lipids Health Dis.

[39] Nozaki T, Sugiyama S, Sugamura K (2010). Prognostic value of endothelial extracellular vesicles in patients with heart failure. Eur J Heart Fail.

[40] Block A, Wheeler C, Persky S (2012). Effects of phosphate binders in moderate CKD. J Am Soc Nephrol.

