# Supplementary material for: RNA-seq analysis of extracellular vesicles from hyperphosphatemia-stimulated endothelial cells provides insight into the mechanism underlying vascular calcification
Source: BMC Nephrol. 2022 May 21;23:192. doi: 10.1186/s12882-022-02823-6 (PMC9123672; doi:10.1186/s12882-022-02823-6)
Supplement: Supplementary file 1 — Additional file 1. [file 12882_2022_2823_MOESM1_ESM.docx]

**Supplementary Materials and Methods**

**Scanning Electron Microscopy**

To confirm the existence of HP-EC-EVs, we used scanning electron microscope (SEM) imaging as described before [1]. First, HP-EC-EVs were isolated from the culture medium after HUVECs incubation with 3 mM [Pi] medium for 48 hours as described above. Then, the samples were fixed by using precooled 1% paraformaldehyde solution for 20 min and placed on the specimen stub and immediately snap frozen in freeze‐dryer. Finally, the dried samples were sputter-coated with gold for 30 seconds and observed under a hitachi SU8100 Scanning Electron Microscope.

**Flow Cytometry Analysis of EC-EVs**

Supernatant from HUVECs treated with Pi for 24 hours or 48 hours was collected to obtain EC-EVs. EC-EVs were identified as we described previously [2,3]. Endothelial-derived EVs were defined as <1μm and annexin V^+^ CD144^+^. The number of EC-EV type per microliter was calculated by Trucount tube [with a precise number of fluorescent beads (48 678) to determine the number of EVs in a sample after accumulation of 10 000 gated events.

**EC-EVs labeling and uptake by VSMCs**

EC-EVs were labeled with the red fluorescent dye CellTracker DiD (AAT Bioquest, Sunnyvale, CA, USA) as described in previous study [4,5]. EC-EVs labeling with CellTracker DiD were performed following the manufacturer’s procedures. EC-EVs from 1.5×10^8^ HUVECs were resuspended in 200 μl PBS with 12 μl/ml diluted CellTracker DiD. After 20 min of incubation at room temperature, VSMCs were incubated with the CellTracker DiD-labeled EC-EVs at 37 ℃ for 20 min, 2 h, 8 h, or 24 h. VSMCs were then washed in 4% formaldehyde in PBS for 20 min. After washing with PBS, nuclei were stained with DAPI (Invitrogen, Carlsbad, CA, USA). The signals were analyzed with a fluorescence microscope.

**In vitro calcification and quantification**

VSMCs were cultured with EC-EVs or 3 mM [Pi] for 7 days. After washing with cold phosphate buffered saline (PBS), cells or aortic rings were treated with 0.6 mmol/l HCl overnight at 4 °C. The calcium content in the HCl supernatant was subjected to colourimetric analysis using a Calcium Assay Kit (BioSino, Beijing, China) and normalized to the protein content. In parallel sets, ALP activity was measured in a colourimetric analysis using an ALP assay kit (BioSino) according to the manufacturer’s instructions.

**Alizarin Red S**

For Alizarin red S staining, VSMCs in 3 cm^2^ dishes were fixed in 4% formaldehyde for 10 min at room temperature, exposed to 2% Alizarin red S (Sigma) for 30 min and washed with 0.2% acetic acid. Positively stained cells showed a reddish/purple color.

**RNA extraction, miRNA sequencing library construction, HTS, bioinformatic analysis**

RNA extraction, miRNA sequencing library construction, HTS, and bioinformatic analysis were performed as described before [6]. Total RNA was extracted using mirVana miRNA Isolation Kit (Ambion) according to the manufacturer’s protocol. Quantitation of total RNA was carried out using the Nanodrop 2000 (Thermo Fisher Scientific Inc., USA). RNA integrity was assessed by Agilent 2100 Bioanalyzer (Agilent Technology, USA).

Approximately 1 μg total RNA of each sample was used for the small RNA library construction using TruSeq Small RNA Sample Prep Kits (Cat. No. RS-200-0012, Illumina, USA.) following the manufacturer’s recommendations. Briefly, total RNA were ligated to adapters at each end. Then the adapter-ligated RNA were reverse transcribed to cDNA and performed PCR amplification. The PCR products ranging from 140–160 bp were isolated and purified as small RNA libraries. Library quality was assessed on the Agilent Bioanalyzer 2100 system using DNA High Sensitivity Chips. The libraries were finally sequenced using the Illumina HiSeq X Ten platform. 150 bp paired-end reads were generated. The small RNA sequencing and analysis for HP-EC-EVs and PBS-EC-EVs were conducted by OE Biotech Co., Ltd. (Shanghai, China). Each group processed three samples.

The basic reads were converted into sequence data (also called raw data/reads) by base calling. Low quality reads were filtered, and the reads with 5’ primer contaminants and poly (A) were removed. The reads without 3’adapter and insert tag, the reads shorter than 15 nt or longer than 41 nt from the raw data were filtered, and the clean reads were obtained.

For primary analysis, the length distribution of the clean sequences in the reference genome was determined. Non-coding RNAs were annotated as rRNAs, tRNAs, small nuclear RNAs (snRNAs), and so on. These RNAs were aligned and then subjected to the Bowtie [7] search against Rfam v.10.1 (http://www.sanger.ac.uk/software/Rfam) [8]. The known miRNAs were identified by aligning against miRBase v22 database (http://www.mirbase.org/) [9], and the known miRNA expression patterns in different samples were analyzed. After that, unannotated reads were analyzed by mirdeep2 [10] to predict novel miRNAs. Based on the hairpin structure of a pre-miRNA and the miRBase database, the corresponding miRNA star sequence and miRNA mature sequence were also identified.

When comparing the differentially expressed miRNA profiles between two groups, fold change and P-values were calculated and used to identify significant differentially expressed miRNAs. Hierarchical clustering was utilized to display the differentially expressed miRNAs. miRNA target prediction was performed by using three database: mirDB, mirWalk, and miranda database [11-13]. To improve prediction accuracy, the overlap of the predicted results from the three programs was considered to represent the final result of predicted target mRNAs (When the miRNAs of HP-EC-EVs_vs PBS-EC-EVs_down and HP-EC-EVs_vs PBS-EC-EVs_up were predicted as target genes, the values of 3 databases including mirDB, mirWalk, and miranda). Venn diagram showed the expression distribution of all the target genes of differentially expressed miRNAs. To reveal correlations among miRNAs, miRNA target genes, and target genes in EC-EVs, miRNA-mRNA networks were constructed using the Cytoscape software manual (http://www.cytoscape.org) [14]. The GO and KEGG pathway analysis were performed based on the differentially expressed genes (DEGs). Our raw data can be found on the BioProject (accession number: PRJNA747859).

**Differential miRNA verification by qRT-PCR**

qRT-PCR was carried out to validate the miRNAs identified by deep sequencing. We chose one significantly up-regulated and 12 significantly down-regulated miRNAs closely associated with vascular calcification. In total, 13 miRNAs were verified by qRT-PCR. The samples were reverse-transcribed into cDNA using the Mir-X™ miRNA First Strand Synthesis Kit (Code No.638315; Takara, Tokyo, Japan) according to the manufacturer's instructions. cDNA was quantified by the Mir-X™ miRNA qRT-PCR SYBR®Kit (Code No. 638316, Takara) using a 25 μL reaction mixture that consisted of 2 μL diluted cDNA, 9 μL ddH_2_O, 0.5 μL ROX Dye (50×), 0.5 μL miRNA-specific primer (10 μL), 0.5 μL mRQ 3′ primer, and 12.5 μL SYBR Advantage Premix (2×). U6 was chosen as the PCR control. The data was analyzed using 2^−ΔΔCT^ method. Primer sequences used for qRT-PCR are listed in **Supplementary Table S1.**

**References**

1. Elhameh S, Alireza N, Reza R, et al, Treatment of human neuroblastoma cell line SH-SY5Y with HSP27 siRNA tagged-exosomes decreased differentiation rate into matureneurons. J Cell Physiol. 2019;234:21005-21013.

2. He Z, Zhang Y, Cao M, et al, Increased phosphatidylserine-exposing microparticles and their originating cells are associated with the coagulation process in patients with IgA nephropathy. Nephrol Dial Transplant. 2016;31:747-59.

3. He Z, Si Y, Jiang T, et al, Phosphotidylserine exposure and neutrophil extracellular traps enhance procoagulant activity in patients with inflammatory bowel disease. Thromb Haemost. 2016;115:738-51.

4. Li S, Zhan J, Wang Y, et al, Exosomes from hyperglycemia-stimulated vascular endothelial cells contain versican that regulate calcification/senescence in vascular smooth muscle cells. Cell Biosci. 2019;9:1.

5. Tian T, Zhu Y, Zhou Y, et al. Exosome uptake through clathrin-mediated endocytosis and macropinocytosis and mediating miR-21 delivery. J Biol Chem. 2014;289:22258–22267.

6. Ren H, Yu X, Shen G, et al. miRNA-seq analysis of human vertebrae provides insight into the mechanism underlying GIOP. Bone. 2019;120:371-386.

7. Langmead B, Trapnell C, Pop M, et al. Ultrafast and memory-efficient alignment of short DNA sequences to the human genome. Genome Biol. 2009;10(3):R25.

8. Griffiths-Jones S, Bateman A, Marshall M, et al. Rfam: an RNA family database. Nucleic Acids Res. 2003;31(1):439-41.

9. Griffiths-Jones S, Saini HK, van Dongen S, et al. miRBase: tools for microRNA genomics. Nucleic Acids Res. 2008;36:D154-8.

10. Friedlander MR, Mackowiak SD, Li N, et al. miRDeep2 accurately identifies known and hundreds of novel microRNA genes in seven animal clades. Nucleic Acids Res. 2012;40:37–52.

11. Wang X. miRDB: a microRNA target prediction and functional annotation database with a wiki interface. RNA. 2018;14(6):1012-7.

12. Dweep H, Sticht C, Pandey P, et al. miRWalk–database: prediction of possible miRNA binding sites by “walking”the genes of three genomes. J Biomed Inform. 2011;44(5):839-47.

13. John B, Enright AJ, Aravin A, et al. Human microRNA targets. PLoS Biol. 2004;2(11):e363.

14. Politano G, Benso A, Savino A, et al. ReNE: a cytoscape plugin for regulatory network enhancement. PLoS One. 2014;9 (12): e115585.
