# Supplementary material for: RNA-seq analysis of extracellular vesicles from hyperphosphatemia-stimulated endothelial cells provides insight into the mechanism underlying vascular calcification
Source: BMC Nephrol. 2022 May 21;23:192. doi: 10.1186/s12882-022-02823-6 (PMC9123672; doi:10.1186/s12882-022-02823-6)
Supplement: Supplementary file 2 — Additional file 2: Table S1. The primer used in QPCR. Table S2. Down-regulated and up-regulated miRNAs. Table S3. KEGG pathways of total differentially expressed miRNAs between HP-EMPs and PBS-EMPs groups. [file 12882_2022_2823_MOESM2_ESM.docx]

**Table S1** The primer used in QPCR.

| **miRNAs** | **Primers** |
| --- | --- |
| hsa-miR-10a-5p | Forward: 5’-UACCCUGUAGAUCCGAAUUUGUG-3’ |
| hsa-miR-10b-5p | Forward: 5’-UACCCUGUAGAACCGAAUUUGUG-3’ |
| hsa-miR-143-3p | Forward: 5’-UGAGAUGAAGCACUGUAGCUC-3’ |
| hsa-miR-193b-5p | Forward: 5’-CGGGGUUUUGAGGGCGAGAUGA-3’ |
| hsa-miR-30a-3p | Forward: 5’-CUUUCAGUCGGAUGUUUGCAGC-3’ |
| hsa-miR-30a-5p | Forward: 5’-UGUAAACAUCCUCGACUGGAAG-3’ |
| hsa-miR-30c-2-3p | Forward: 5’-CUGGGAGAAGGCUGUUUACUCU-3’ |
| hsa-miR-3182 | Forward: 5’-GCUUCUGUAGUGUAGUC-3’ |
| hsa-miR-365a-5p | Forward: 5’-AGGGACUUUUGGGGGCAGAUGUG-3’ |
| hsa-miR-486-5p | Forward: 5’-UCCUGUACUGAGCUGCCCCGAG-3’ |
| hsa-miR-7706 | Forward: 5’-UGAAGCGCCUGUGCUCUGCCGAGA-3’ |
| hsa-miR-941 | Forward: 5’-CACCCGGCUGUGUGCACAUGUGC-3’ |
| hsa-miR-99b-5p | Forward: 5’-CACCCGUAGAACCGACCUUGCG-3’ |

**Table S2** Down-regulated and up-regulated miRNAs.

| **Mature ID** | **PRE-ID** | **Fold change** | **P value** | **Expression types** | **Sequence** |
| --- | --- | --- | --- | --- | --- |
| hsa-miR-10a-5p | hsa-miR-10a | 0.137 | 0.0010 | Down | TACCCTGTAGATCCGAATTTGTG |
| hsa-miR-10b-5p | hsa-miR-10b | 0.223 | 0.0204 | Down | TACCCTGTAGAACCGAATTTGTG |
| hsa-miR-143-3p | hsa-miR-143 | 0.176 | 0.0199 | Down | TGAGATGAAGCACTGTAGCTC |
| hsa-miR-193b-5p | hsa-miR-193b | 0.077 | 0.0008 | Down | CGGGGTTTTGAGGGCGAGATGA |
| hsa-miR-30a-3p | hsa-miR-30a | 0.257 | 0.0436 | Down | CTTTCAGTCGGATGTTTGCAGC |
| hsa-miR-30a-5p | hsa-miR-30a | 0.352 | 0.049164 | Down | TGTAAACATCCTCGACTGGAAG |
| hsa-miR-30c-2-3p | hsa-miR-30c-2 | 0.148 | 0.0056 | Down | CTGGGAGAAGGCTGTTTACTCT |
| hsa-miR-3182 | hsa-miR-3182 | 12.634 | 0.0356 | Up | GCTTCTGTAGTGTAGTC |
| hsa-miR-365a-5p | hsa-miR-365a | 0.1438 | 0.0310 | Down | AGGGACTTTTGGGGGCAGATGTG |
| hsa-miR-486-5p | hsa-miR-486 | 0.263 | 0.0493 | Down | TCCTGTACTGAGCTGCCCCGAG |
| hsa-miR-7706 | hsa-miR-7706 | 0.089 | 0.0275 | Down | TGAAGCGCCTGTGCTCTGCCGAGA |
| hsa-miR-941 | hsa-miR-941 | 0.210 | 0.0453 | Down | CACCCGGCTGTGTGCACATGTGC |
| hsa-miR-99b-5p | hsa-miR-99b | 0.199 | 0.0173 | Down | CACCCGTAGAACCGACCTTGCG |
| hsa-miR-novel-Chr12_15948 | hsa-miR-novel-Chr12_15948 | 11.780 | 0.0143 | Up | CTGTTGAAACTGAAGATG |
| hsa-miR-novel-Chr1_1205 | hsa-miR-novel-Chr1_1205 | 24.393 | 0.0304 | Up | CTGGAGCTGGAGTCAGTC |
| hsa-miR-novel-Chr2_2716 | hsa-miR-novel-Chr2_2716 | 13.248 | 0.0001 | Up | ATGGCAGCAGACTTGATG |
| hsa-miR-novel-Chr14_17587 | hsa-miR-novel-Chr14_17587 | Inf | 0.0392 | Up | AGGTTGGAAGAGTAAGTG |
| hsa-miR-novel-Chr1_887 | hsa-miR-novel-Chr1_887 | 18.986 | 0.0303 | Up | GGAAGGAGACAGTGGGTG |
| hsa-miR-novel-Chr17_19401 | hsa-miR-novel-Chr17_19401 | 4.650 | 0.0112 | Up | AGAGGGACGGCCGGGGGT |
| hsa-miR-novel-Chr6_9236 | hsa-miR-novel-Chr6_9236 | 3.320 | 0.0479 | Up | GGAGTTCTGGACTGTAGTG |
| hsa-miR-novel-Chr9_12439＞hsa-miR-novel-Chr9_12440 | hsa-miR-novel-Chr9_12439＞hsa-miR-novel-Chr9_12440 | 5.627 | 0.0401 | Up | GCTGGAAAGGCGGCCGCC |
| hsa-miR-novel-Chr14_17089＞hsa-miR-novel-Chr14_17090 | hsa-miR-novel-Chr14_17089＞hsa-miR-novel-Chr14_17090 | 4.218 | 0.0127 | Up | GAGTTCGGGGCTGTAGTG |
| hsa-miR-novel-ChrX_24044 | hsa-miR-novel-ChrX_24044 | Inf | 1.42E-06 | Up | GAGTTCTGGGCTGTAGTA |
| hsa-miR-novel-Chr10_13624 | hsa-miR-novel-Chr10_13624 | 3.154 | 0.0376 | Up | GGGTTCTGGGCTGTAGTG |
| hsa-miR-novel-Chr1_1405 | hsa-miR-novel-Chr1_1405 | Inf | 0.0004 | Up | AGTTTCTGTTGCTGGTTC |
| hsa-miR-novel-Chr12_15143 | hsa-miR-novel-Chr12_15143 | 5.798 | 0.0009 | Up | AGTTCTGGGCTGTAGTGAGCTA |
| hsa-miR-novel-Chr11_14558 | hsa-miR-novel-Chr11_14558 | 0.042 | 0.0249 | Down | GAGTTCTTGGCTGTAGTGTGCT |
| hsa-miR-novel-Chr7_10732 | hsa-miR-novel-Chr7_10732 | 15.968 | 0.0043 | Up | AGGACTGATGGCTGGGGGG |
| hsa-miR-novel-Chr8_10922 | hsa-miR-novel-Chr8_10922 | 11.578 | 0.0003 | Up | GGAGTTCTGGGCTGTAGGG |
| hsa-miR-novel-Chr14_17649 | hsa-miR-novel-Chr14_17649 | 12.767 | 0.0001 | Up | AAGAGGCTGGAGAGGACG |
| hsa-miR-novel-Chr18_19949 | hsa-miR-novel-Chr18_19949 | 7.209 | 0.0039 | Up | GAGTTCTGGGCTGTAGCA |
| hsa-miR-novel-Chr20_21071 | hsa-miR-novel-Chr20_21071 | Inf | 0.0053 | Up | GGAAGGTGGAGAAGCTGACC |
| hsa-miR-novel-Chr9_12684 | hsa-miR-novel-Chr9_12684 | 6.187 | 0.0057 | Up | CTCAAGAGGCTGAAGAGGCC |
| hsa-miR-novel-Chr2_3698 | hsa-miR-novel-Chr2_3698 | 5.847 | 0.0036 | Up | GAGTTCTGGGCTGTAGAGCGCT |
| hsa-miR-novel-Chr12_15908 | hsa-miR-novel-Chr12_15908 | Inf | 3.38E-06 | Up | GAGTTCTTGGCTGTAGTG |
| hsa-miR-novel-Chr11_14340 | hsa-miR-novel-Chr11_14340 | 0.243 | 0.0463 | Down | AGGGAGACTTTGAAGCTG |
| hsa-miR-novel-Chr3_4097 | hsa-miR-novel-Chr3_4097 | 14.648 | 0.0004 | Up | GATTTCTGGGCTGTAGTG |
| hsa-miR-novel-Chr4_5977>hsa-miR-novel-Chr9_12862 | hsa-miR-novel-Chr4_5977>hsa-miR-novel-Chr9_12862 | 4.529 | 0.0094 | Up | GATTTGGTCGTGGACGTGGTC |
| hsa-miR-novel-Chr1_1551 | hsa-miR-novel-Chr1_1551 | 0.063 | 0.0217 | Down | GTGAAAGGGATCCTGAAA |
| hsa-miR-novel-Chr21_22407 | hsa-miR-novel-Chr21_22407 | Inf | 0.0467 | Up | TCAGTGGGGCCACGAGCTG |
| hsa-miR-novel-Chr6_9152 | hsa-miR-novel-Chr6_9152 | Inf | 0.0082 | Up | CAGTGCTTGGACGGAACCCG |
| hsa-miR-novel-Chr1_1198 | hsa-miR-novel-Chr1_1198 | 2.640 | 0.0318 | Up | GAGTTCTAGGCTGTAGTG |
| hsa-miR-novel-Chr3_5131＞hsa-miR-novel-Chr3_5132 | hsa-miR-novel-Chr3_5131＞hsa-miR-novel-Chr3_5132 | 8.251 | 0.0172 | Up | CTCAAGAGGCTGAAGAGA |
| hsa-miR-novel-Chr3_4787 | hsa-miR-novel-Chr3_4787 | 19.174 | 5.80E-05 | Up | GATGACTGGGGTGAAGTT |
| hsa-miR-novel-Chr2_3293 | hsa-miR-novel-Chr2_3293 | 16.993 | 0.0035 | Up | GTAGTGAAGCGGTGGAAAG |
| hsa-miR-novel-Chr5_7115＞hsa-miR-novel-Chr1_1380 | hsa-miR-novel-Chr5_7115＞hsa-miR-novel-Chr1_1380 | 91.625 | 0.0002 | Up | AGGACTGAGGACTGGTGG |
| hsa-miR-novel-Chr10_13642 | hsa-miR-novel-Chr10_13642 | 6.587 | 0.0022 | Up | CCCAAGGCAGGACTGATG |

**Table S3** KEGG pathways of total differentially expressed miRNAs between HP-EMPs and PBS-EMPs groups.

| **Pathway ID** | **Definition** | **Fisher-P value** | **Enrichment score** |
| --- | --- | --- | --- |
| path:hsa04136 | Autophagy - other | 6.75E-33 | 4.708866515 |
| path:hsa00534 | Glycosaminoglycan biosynthesis-heparan sulfate/heparin | 5.09E-09 | 3.465242794 |
| path:hsa01040 | Biosynthesis of unsaturated fatty acids | 8.60E-09 | 3.306402925 |
| path:hsa00062 | Fatty acid elongation | 8.85E-09 | 3.089016434 |
| path:hsa04970 | Salivary secretion | 1.19E-28 | 3.082454346 |
| path:hsa04972 | Pancreatic secretion | 3.45E-25 | 2.971593468 |
| path:hsa04211 | Longevity regulating pathway | 2.43E-26 | 2.734376135 |
| path:hsa04370 | VEGF signaling pathway | 6.23E-13 | 2.533958793 |
| path:hsa04140 | Autophagy - animal | 8.90E-27 | 2.42296661 |
| path:hsa04640 | Hematopoietic cell lineage | 3.14E-10 | 2.164651668 |
| path:hsa00310 | Lysine degradation | 5.56E-09 | 2.12842864 |
| path:hsa04924 | Renin secretion | 2.62E-09 | 2.07666564 |
| path:hsa05210 | Colorectal cancer | 4.34E-10 | 1.991806868 |
| path:hsa05231 | Choline metabolism in cancer | 1.80E-08 | 1.846273701 |
| path:hsa04932 | Non-alcoholic fatty liver disease (NAFLD) | 3.66E-08 | 1.805011743 |
| path:hsa04022 | cGMP-PKG signaling pathway | 1.27E-10 | 1.772756218 |
| path:hsa04270 | Vascular smooth muscle contraction | 1.31E-08 | 1.748684881 |
| path:hsa04925 | Aldosterone synthesis and secretion | 3.45E-07 | 1.717938165 |
| path:hsa05170 | Human immunodeficiency virus 1 infection | 7.17E-11 | 1.716376225 |
| path:hsa04070 | Phosphatidylinositol signaling system | 5.28E-08 | 1.71213432 |
