# Supplementary figures and images for: RNA-seq analysis of extracellular vesicles from hyperphosphatemia-stimulated endothelial cells provides insight into the mechanism underlying vascular calcification
Source: BMC Nephrol. 2022 May 21;23:192. doi: 10.1186/s12882-022-02823-6 (PMC9123672; doi:10.1186/s12882-022-02823-6)

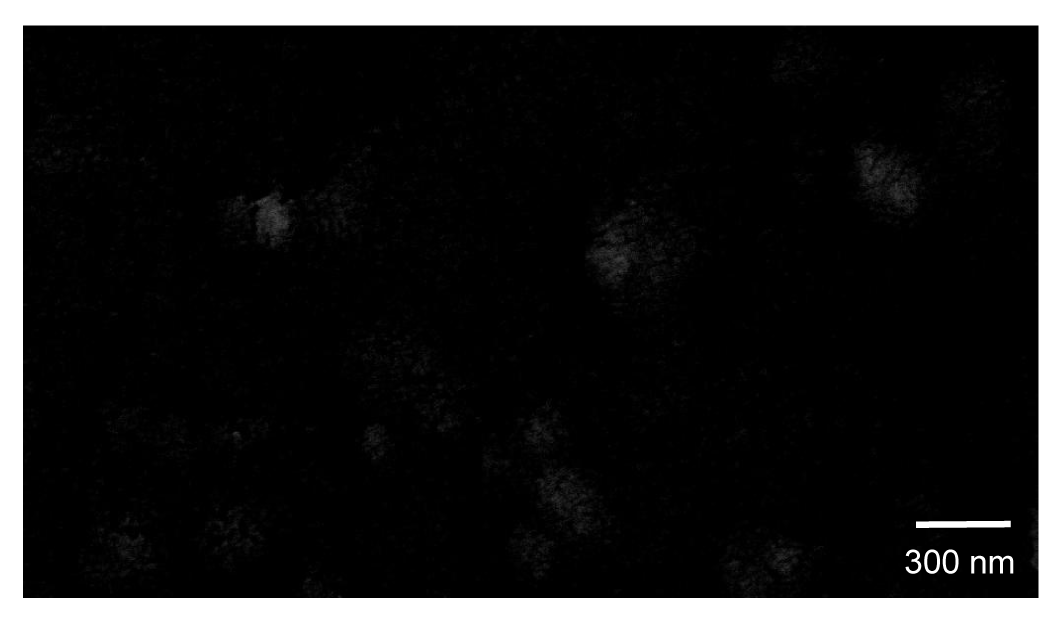

Supplement: Supplementary file 3 — Additional file 3. [file 12882_2022_2823_MOESM3_ESM.tif]

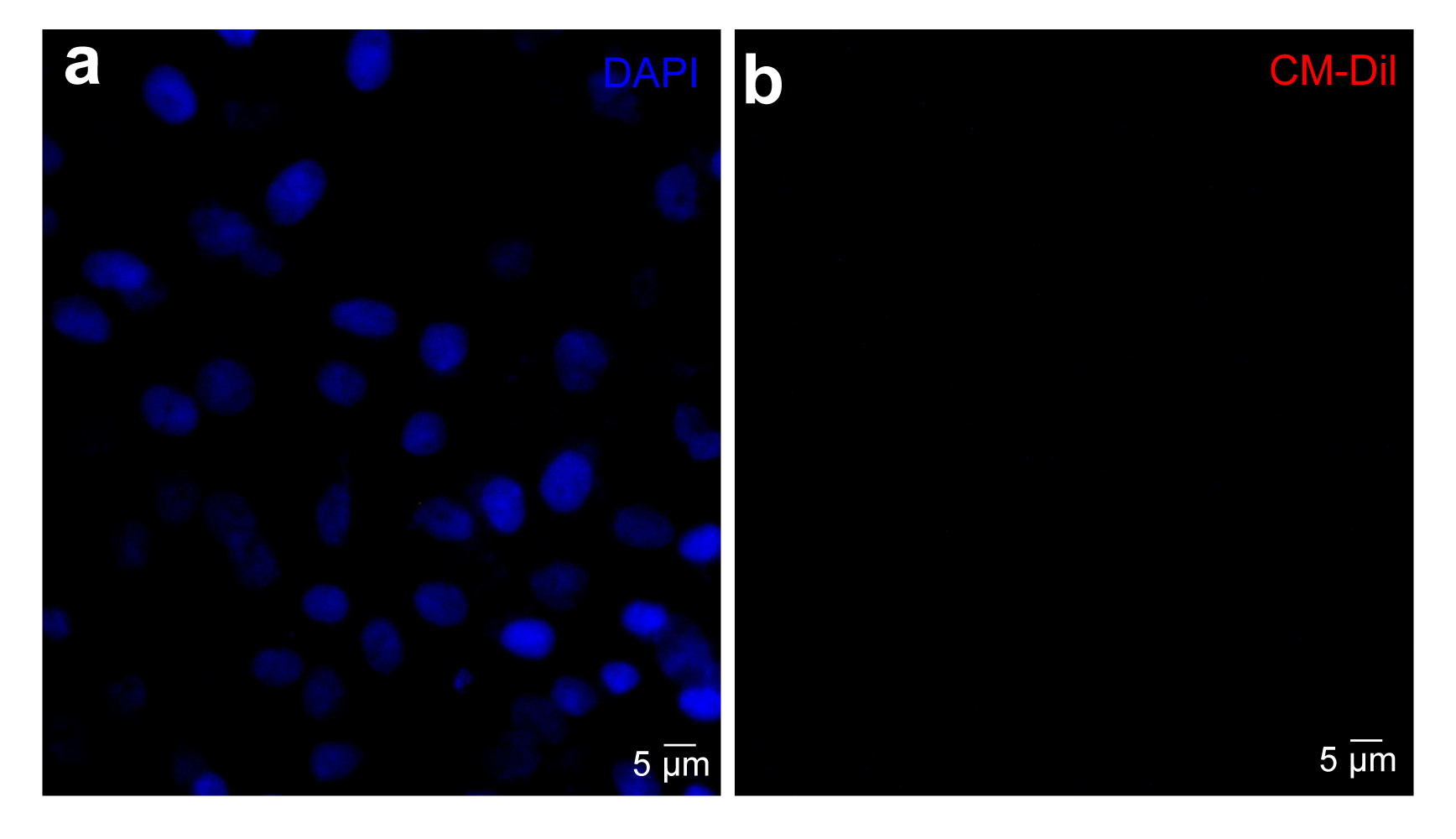

Supplement: Supplementary file 4 — Additional file 4. [file 12882_2022_2823_MOESM4_ESM.tif]
